# Fatigue in patients with chronic disease: results from the population-based Lifelines Cohort Study

**DOI:** 10.1038/s41598-021-00337-z

**Published:** 2021-10-25

**Authors:** Yvonne M. J. Goërtz, Annemarie M. J. Braamse, Martijn A. Spruit, Daisy J. A. Janssen, Zjala Ebadi, Maarten Van Herck, Chris Burtin, Jeannette B. Peters, Mirjam A. G. Sprangers, Femke Lamers, Jos W. R. Twisk, Melissa S. Y. Thong, Jan H. Vercoulen, Suzanne E. Geerlings, Anouk W. Vaes, Rosanne J. H. C. G. Beijers, Martijn van Beers, Annemie M. W. J. Schols, Judith G. M. Rosmalen, Hans Knoop

**Affiliations:** 1 Department of Research and Development, Ciro, Hornerheide 1, 6085 NM Horn, The Netherlands; 2 NUTRIM School of Nutrition and Translational Research in Metabolism, Maastricht, The Netherlands; 3 Department of Respiratory Medicine, Maastricht University Medical Centre (MUMC+), Maastricht, The Netherlands; 4 Department of Medical Psychology, Amsterdam Public Health Research Institute, Amsterdam University Medical Centers, University of Amsterdam, Amsterdam, The Netherlands; 5 Department of Health Services Research, Care and Public Health Research Institute, Faculty of Health, Medicine and Life Sciences, Maastricht University, Maastricht, The Netherlands; 6 Department of Medical Psychology, Radboud Institute for Health Sciences, Radboud university medical center, Nijmegen, The Netherlands; 7 REVAL-Rehabilitation Research Center, BIOMED-Biomedical Research Institute, Faculty of Rehabilitation Sciences, Hasselt University, Diepenbeek, Belgium; 8 Department of Lung Diseases, Radboud Institute for Health Sciences, Radboud university medical center, Nijmegen, The Netherlands; 9 Department of Psychiatry, Amsterdam Public Health Research Institute, Amsterdam University Medical Centers, Vrije Universiteit Amsterdam, Amsterdam, The Netherlands; 10 Department of Epidemiology and Biostatistics, Amsterdam Public Health Research Institute, Amsterdam University Medical Centers, Vrije Universiteit Amsterdam, Amsterdam, The Netherlands; 11 Department of Internal Medicine, Amsterdam University Medical Centers, University of Amsterdam, Amsterdam, The Netherlands; 12 Departments of Psychiatry and Internal Medicine, University Medical Center Groningen, University of Groningen, Groningen, The Netherlands

**Keywords:** Fatigue, Comorbidities

## Abstract

(1) To evaluate the prevalence of severe and chronic fatigue in subjects with and without chronic disease; (2) to assess to which extent multi-morbidity contributes to severe and chronic fatigue; and (3) to identify predisposing and associated factors for severe and chronic fatigue and whether these are disease-specific, trans-diagnostic, or generic. The Dutch Lifelines cohort was used, including 78,363 subjects with (n = 31,039, 53 ± 12 years, 33% male) and without (n = 47,324, 48 ± 12 years, 46% male) ≥ 1 of 23 chronic diseases. Fatigue was assessed with the Checklist Individual Strength-Fatigue. Compared to participants without a chronic disease, a higher proportion of participants with ≥ 1 chronic disease were severely (23% versus 15%, *p* < 0.001) and chronically (17% versus 10%, *p* < 0.001) fatigued. The odds of having severe fatigue (OR [95% CI]) increased from 1.6 [1.5–1.7] with one chronic disease to 5.5 [4.5–6.7] with four chronic diseases; for chronic fatigue from 1.5 [1.5–1.6] to 4.9 [3.9–6.1]. Multiple trans-diagnostic predisposing and associated factors of fatigue were found, explaining 26% of variance in fatigue in chronic disease. Severe and chronic fatigue are highly prevalent in chronic diseases. Multi-morbidity increases the odds of having severe and chronic fatigue. Several trans-diagnostic factors were associated with fatigue, providing a rationale for a trans-diagnostic approach.

## Introduction

For most people, fatigue is an everyday experience. It becomes a symptom when it is an overwhelming feeling of exhaustion that interferes with the ability to function and perform activities^1^. Severe fatigue that persists longer than 6 months is defined as chronic^2^. Severe and chronic fatigue is a symptom of many non-communicable chronic diseases e.g. Parkinson’s disease (PD)^3^, chronic heart failure (CHF)^4^, chronic obstructive pulmonary disease (COPD)^5^, type I diabetes mellitus (T1DM)^6^, rheumatoid arthritis (RA)^7^, and inflammatory bowel disease^8^. Fatigue is associated with functional impairment and often reported by patients to be one of the most burdensome and challenging aspects of their disease^9,10^. Thus far, fatigue is mostly studied in the context of a single chronic condition. However, the proportion of patients living with two or more chronic diseases concurrently (i.e., multi-morbidity) rises^11,12^. This complicates our understanding of the relationship between chronic disease and fatigue.

Research comparing the prevalence of fatigue in people with and without a specific chronic disease generally found that fatigue is more prevalent in patients with a chronic disease^5,8,13–15^. This suggests that fatigue is linked to a specific chronic disease. Moreover, since prevalence rates of severe fatigue seem to vary across chronic diseases^16^, it is often assumed that fatigue is a disease-specific symptom. However, the relationship between traditional markers of disease (severity and activity) and fatigue is generally poor or even non-existent. For instance, 40% of patients with T1DM experience chronic fatigue, but fatigue is weakly associated with abnormal glucose parameters^6^. A similar lack of a relationship between markers of disease- and fatigue severity, has been reported in patients with multiple sclerosis (MS)^14^, COPD^5^, asthma^17^, systemic lupus erythematosus (SLE)^18^, RA^19^, idiopathic pulmonary fibrosis and sarcoidosis^20^. Hence, it seems plausible that other than the specific disease severity factors may be involved.

In identifying the factors that contribute to fatigue in chronic disease, two types can be distinguished: (1) time-independent or pre-morbid predisposing factors, that are not likely to change over time, and (2) time-dependent associated or maintaining factors, which may change over time. Knowledge about pre-morbid predisposing factors (hereafter called ‘predisposing factors’) will help identify patients at risk for developing clinically relevant levels of fatigue, while time-dependent associated factors (hereafter called ‘associated factors’) can be addressed in interventions that can reduce fatigue or help patients to manage fatigue. Predisposing socio-demographic characteristics such as a lower education^21–23^ and being female^21,23^ were found to be related to higher levels of fatigue in specific chronic diseases. Furthermore, certain personality traits are assumed to increase a person’s risk of becoming severely fatigued in response to a chronic disease. Indeed, there is evidence from research in patients with MS that there is a link between neuroticism and fatigue^24–26^. Besides these predisposing factors, modifiable factors such as depressed mood and anxiety may be associated with, or maintain, fatigue in specific chronic diseases^6,27–30^. Also, lower physical activity levels^31–33^, sleep disturbances^7^, lower body mass index (BMI)^34^, hyper- or hypotension^35,36^, pain^6,28,37,38^, and smoking status^39,40^ have been identified as factors possibly contributing to fatigue in specific chronic diseases.

Thus, fatigue in patients with a chronic illness is a complex symptom that likely involves various predisposing and associated factors. Some of the factors seem to be associated with multiple chronic diseases. Nevertheless, the above-mentioned factors have all been studied in a disease-specific context. To date, studies seldom have examined fatigue across a wide range of chronic diseases simultaneously. Only one study investigated fatigue across 15 chronic diseases, combining data from 15 clinical studies^16^. The results indicated that factors associated with fatigue seem mostly trans-diagnostic. Limitations however included that their sample might have been biased because of an over- or underrepresentation of fatigue cases, as data from 15 clinical studies were combined, (often) specifically designed to study fatigue. In addition, multi-morbidity and some major chronic diseases such as cardiovascular disease and chronic lung disease were not considered in that study, and a control group without chronic disease was lacking.

To date, fatigue levels have never been assessed within one large, longitudinal cohort from the general population involving presumably healthy subjects as well as patients with various chronic diseases. Such a study would enable the comparison of fatigue prevalence rates of patients with a variety of conditions and a population without chronic disease. A better understanding of the factors underlying fatigue across a wide range of conditions and healthy subjects, and knowing whether these are disease-specific, trans-diagnostic (similar for multiple chronic diseases) or generic (similar for persons with and without a chronic disease), may optimize and accelerate the development of interventions for fatigue to improve daily functioning of patients with a chronic disease. Moving away from a disease-specific focus to a trans-diagnostic approach also supports the complexity of multi-morbid chronic disease management, as disease-centered treatments will not fully address the comprehensive needs of patients with multiple chronic diseases^41^.

The objectives of the present study were: (1) to evaluate the prevalence of severe and chronic fatigue in subjects with and without chronic disease; (2) to assess the extent to which multi-morbidity contributes to severe and chronic fatigue; and (3) to identify possible predisposing and associated factors for severe and chronic fatigue and whether these are disease-specific, trans-diagnostic, or generic.

## Methods

### Study design and participants

The current study used data from the Lifelines Cohort Study^42,43^. Lifelines is an ongoing multi-disciplinary prospective population-based cohort study examining in a unique three-generation design the health and health-related behaviours of 167,729 persons living in the North of The Netherlands. It employs a broad range of investigative procedures in assessing the biomedical, socio-demographic, behavioural, physical and psychological factors which contribute to the health and disease of the general population, with special focus on multi-morbidity and complex genetics. The participants were recruited between 2006 and 2013, through general practitioners and self-enrollment. Participants who were unable to understand the Dutch language, were not able to fill in questionnaires, not able to visit the general practitioner, had severe mental illness (i.e. not fully capable to make rational decisions), or who had limited life expectancy (< 5 years) due to severe illness were not considered eligible. Every 5 years, participants visit a Lifelines research site for the collection of biological materials and a comprehensive physical assessment. In addition, participants are asked to fill out an extensive set of questionnaires, including questions on medical history, socioeconomic status, psychological status, environmental factors, and lifestyle. In-between these 5-year assessments, once every 1.5 years a follow-up questionnaire is administered. The Lifelines Cohort Study was conducted according to the principles of the Declaration of Helsinki and approved by the Medical Ethics Committee of the University Medical Center Groningen, the Netherlands (number 2007/152). No additional ethical approval is needed to request data collected within the regular protocol of Lifelines. All participants signed an informed consent. A detailed description of the Lifelines Cohort Study has been published elsewhere^42–44^.

### Procedures

The main outcome parameter fatigue as well as the time-dependent associated factors were measured at the first follow-up measurement, which was approximately 5 years after the baseline assessment. The predisposing factors and the presence of a chronic illness were evaluated at the baseline assessment, assuming that these are time-independent. The associated and predisposing factors were selected based on evidence from existing literature, which identified these factors as likely contributors to fatigue in specific chronic diseases. Participants were included in the current study if they had provided information on the main outcome parameter fatigue and were 18 years or older.

### Measures

#### Fatigue severity

Fatigue severity was measured using the Checklist Individual Strength (CIS)^45^. The CIS consists of 20 items that measure four aspects of fatigue: fatigue severity, problems with concentrating, reduction in motivation, and reduced physical activity level. The current study reports on fatigue severity using the subscale fatigue severity (CIS-Fatigue). The CIS-Fatigue consists of eight items scored on a seven-point Likert scale. The score ranges from 8 to 56, with higher scores indicating more severe fatigue. Severe (i.e. clinically relevant) fatigue is indicated by a CIS-Fatigue score ≥ 35 points, a validated cut-off score^46^. Chronic fatigue was defined as severe fatigue (CIS-Fatigue score ≥ 35 points) lasting at least 6 months according to self-report. Participants who experienced severe fatigue but did not report their fatigue duration were omitted from the analyses on chronic fatigue. The CIS is a standardized and validated instrument that has been used in healthy subjects^46–48^, and among various patient populations^5,17,49,50^.

#### Self-reported chronic disease(s)

Participants were asked to self-report the chronic diseases they have from a pre-defined list of 23 chronic medical conditions. The 23 chronic diseases were carefully selected from a broader list of chronic diseases by the authors of the current manuscript based on their non-self-limiting nature, the association with persistent and recurring health problems, and a duration in months and years, not days and weeks^51^. The diseases were clustered in 9 classes of chronic somatic conditions: neurological diseases (migraine, epilepsy, MS, PD, stroke); liver diseases (hepatitis, liver cirrhosis); blood disease (blood clotting disorder); endocrine and metabolic diseases (hypothyroidism or hyperthyroidism, diabetes mellitus type 1 and/or 2); circulatory diseases (thrombosis, myocardial infarction, heart valve problems, pulmonary embolism, CHF, balloon angioplasty and/or bypass surgery); respiratory disease (chronic inflammation of the throat and/or nasal cavity, COPD); inflammatory bowel disease (ulcerative colitis, Crohn’s disease); rheumatic diseases (osteoarthritis, RA); and kidney disease^52^.

#### Possible predisposing factors of fatigue

##### Sociodemographic factors

Sex and education level were assessed with a questionnaire. Education level was divided into low (lower secondary education or less), middle (upper secondary education), and high education (tertiary education).

##### Personality traits

Four facets of neuroticism were measured using the Revised NEO Personality Inventory (NEO PI-R)^53^. Facets of neuroticism included in the Lifelines questionnaire were: anger/hostility, self-consciousness, impulsivity, and vulnerability. Each facet is assessed with eight items, scored on a five-point Likert scale that ranges from strongly disagree to strongly agree.

#### Possible associated factors of fatigue

##### Sociodemographic factors

Self-report questionnaires were used to gather data on age, household composition (single person household, a household with two persons with or without children, a single parent household, and another composition), partner status (yes/no), number of people living in the household, and current employment status (working ≥ 12 h per week or less)^54^.

##### Anthropometry

BMI, waist circumference, resting heart rate in beats and blood pressure measurements (diastolic blood pressure (DBP) and systolic blood pressure (SBP)) were assessed by a research assistant using a standardized protocol^43^.

##### Lifestyle factors

Lifestyle factors included current smoking status (yes/no) and being involved in leisure-time sports activities (yes/no). Sports participation was assessed with the item “sports participation” of the Short Questionnaire to Assess Health-enhancing physical activity (SQUASH)^55^.

##### Mental health disorders

Depressive disorder (depressive disorder and dysthymic mood) and anxiety disorder (panic disorder with and without agoraphobia, agoraphobia without panic disorder, social phobia, and generalized anxiety disorder) were assessed using the self-report version of the Mini-International Neuropsychiatric Interview (M.I.N.I., version 5.0.0)^56^.

##### Pain

A dichotomous variable (yes/no) was created based on one question (e.g. *to what extent did your locomotor apparatus pain hamper your normal activities in the past 6 months?)* indicating whether bodily pain (much or very much) hampered individuals in performing their normal activities^44^.

### Statistical analyses

Statistical analyses were performed using SPSS (V.25.0 for Windows, Chicago, IL, USA).

#### Prevalence

Descriptive statistics and the prevalence rates of fatigue (continuous variable), severe (CIS-Fatigue score ≥ 35, dichotomous variable), and chronic (CIS-Fatigue score ≥ 35 lasting ≥ 6 months, dichotomous variable) fatigue for participants with and without a chronic disease were reported as mean and standard deviation, median and interquartile range, or frequency and percentage, as appropriate.

#### Impact of multi-morbidity on the likelihood of experiencing severe and chronic fatigue

An unadjusted and adjusted (corrected for age and sex^57^) logistic regression was performed to determine the likelihood that participants with one to four chronic diseases were severely and chronically fatigued, compared with persons without a chronic disease.

#### Predisposing and associated factors of fatigue

Analyses of variance (ANOVA) were carried out to investigate whether the possible predisposing and associated factors of fatigue in chronic disease are disease-specific or trans-diagnostic, or generic. The following analyses were performed: (1) a model with only the main effect of chronic disease (model A), (2) a model with the main effect of chronic disease and the potentially predisposing and associated factors (model B), and (3) a model with the same main effects, but also with the interaction effects between chronic disease and the predisposing and associated factors on fatigue severity (model C). Fatigue was entered as a continuous variable. In the disease-specific *versus* trans-diagnostic model the variable “chronic disease” was entered as a categorical variable with 15 categories (each category reflecting a specific chronic disease with no intrinsic order: COPD, blood clotting disorder, stroke, angioplasty and/or bypass surgery, ulcerative colitis, thrombosis, heart valve problems, hepatitis, epilepsy, RA, diabetes mellitus type 1 and/or 2, hypothyroidism or hyperthyroidism, chronic inflammation of throat and/or nasal cavity, osteoarthritis, migraine). In the trans-diagnostic *versus* generic model (i.e., also associated with fatigue in subjects without chronic disease) the categorical variable “chronic disease” consisted of two categories (coded as: 0 = no chronic disease and 1 = single-morbidity). Of note, only participants with single-morbidity and chronic diseases reported by ≥ 150 participants were included in the ANOVA analyses. MS, PD, liver cirrhosis, Crohn’s disease, CHF, myocardial infarction, pulmonary embolism, and kidney disease were therefore excluded as there were less than 150 participants who suffered from these diseases. In case of a significant interaction effect, a simple linear regression was performed as post-hoc test to analyze the effect of the possible predisposing and associated variable on the fatigue severity for a specific chronic disease (the disease-specific *versus* trans-diagnostic model) or for subjects with and without a chronic disease in general (the trans-diagnostic *versus* generic model).

In addition, to explore the relation between the factors and clinically relevant levels of fatigue (severe and chronic fatigue) logistic regression analyses were performed. The logistic regression analyses did not include interaction effects. For the logistic regression models and ANOVA, the predisposing and associated variables were checked for multicollinearity by inspecting the correlation coefficients. Waist circumference and SBP were left out of the analysis since they highly correlated (r > 0.7, p < 0.05) with BMI (r = 0.83) and DBP (r = 0.72). The level of significance was set at < 0.05.

## Results

Between 2006 and 2013, 167,729 subjects were registered in the Lifelines Cohort Study, of whom 78,363 provided information on fatigue during the first follow-up measurement. Of note, 354 participants with a chronic disease and 314 without a chronic disease who experienced severe fatigue did not report their fatigue duration and were therefore omitted from the analyses on chronic fatigue. Forty percent of the total sample (n = 31,039) reported one or more of the 23 chronic diseases. Participants with one or more chronic diseases were on average 5 years older, more often female, less often completed tertiary education, less often currently employed, lived together with fewer people, had a higher BMI, a slightly higher resting heart rate, higher SBP, and a larger waist circumference. Moreover, participants with a chronic disease were less often involved in leisure-time sports activities, more often depressed and anxious, experienced substantially more bodily pain that hampers in performing activities, and scored higher on the neuroticism facets of anger/hostility, self-consciousness, impulsivity, and vulnerability. The proportion of participants that was smoking was higher in the group without chronic disease. The groups were comparable in terms of diastolic blood pressure and having a partner (Table 1). Of the 31,039 participants with one or more chronic diseases, the majority (72%, n = 22,293) had single-morbidity. See Supplemental Fig. 1 for the prevalence of chronic diseases in the sample.

**Table 1:** Baseline characteristics of participants with and without a chronic disease (n = 78,363).

	No chronic illness (n = 47,324)	Chronic illness (n = 31,039)
**Predisposing factors**		
Sociodemographic factors		
Male, n (%)	21,965 (46.4)	10,129 (32.6)
Education, n (%)		
Low	6607 (14.2)	5609 (18.6)
Middle	24,646 (53.1)	16,521 (54.7)
High	15,134 (32.6)	8090 (26.8)
Personality traits		
Neuroticism facets of anger/hostility, self-consciousness, impulsivity, and vulnerability, mean ± SD	19.5 ± 3.1	20.0 ± 3.2
**Associated factors**		
Sociodemographic factors		
Age, mean ± SD	48.2 ± 12.3	53.2 ± 12.2
Household composition, n (%)		
Single person household	4471 (10.9)	3383 (12.8)
Couple household without children	13,785 (33.5)	11,095 (42.0)
Couple household with children	20,156 (49.0)	10,453 (39.6)
Single parent	1349 (3.3)	965 (3.7)
Other	1374 (3.3)	502 (1.9)
Partner, n (%)	35,936 (82.9)	23,572 (82.6)
Currently employed (≥ 12 h/week), n (%)	32,315 (74.9)	16,817 (59.3)
Nr. of people living in the house (median (IQR))	3 (2–4)	2 (2–4)
Anthropometry		
BMI, mean ± SD	25.8 ± 4.0	26.6 ± 4.6
Waist circumference, mean ± SD	89.6 ± 12.1	91.5 ± 12.9
Diastolic blood pressure, mean ± SD	74.2 ± 9.5	74.2 ± 9.4
Systolic blood pressure, mean ± SD	128.1 ± 16.1	130.1 ± 16.8
Resting heart rate, mean ± SD	68.4 ± 11.1	69.5 ± 11.2
Lifestyle		
Currently smoking, n (%)	7703 (16.3)	4608 (14.9)
Currently engaged in leisure-time sports activities, n (%)	24,317 (56.0)	14,256 (49.8)
Mental health		
Current depressive disorder, n (%)	1245 (3.2)	1291 (5.2)
Current anxiety disorder, n (%)	2652 (6.9)	2425 (9.8)
Pain		
Bodily pain that hampers in performing normal activities, n (%)	1307 (2.8)	1915 (6.2)

*n* number; *SD* standard deviation, *IQR* interquartile range, *BMI* body mass index.

### Prevalence of severe and chronic fatigue in the general population: with and without chronic disease

**Figure 1:**
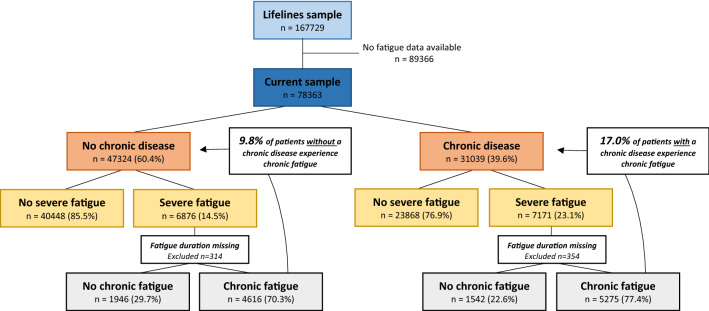
Prevalence of severe and chronic fatigue in participants with and without a chronic disease. Of note, participants with severe fatigue who did not report their fatigue duration were omitted from the analyses on chronic fatigue, hence the smaller sample size of chronic fatigue versus severe fatigue.

Of the 78,363 participants 18% experienced severe fatigue and 13% experienced chronic fatigue. Overall, participants with one or more chronic diseases had a higher mean CIS-Fatigue score (24.8 ± 12.2 versus 21.4 ± 11.1 points, *p* < 0.001) and more often reported severe (23% versus 15%, *p* < 0.001) and chronic (17% versus 10%, *p* < 0.001) fatigue, compared to participants without a chronic disease (Fig. 1).

### Difference in prevalence rate of severe and chronic fatigue among chronic diseases

**Figure 2:**
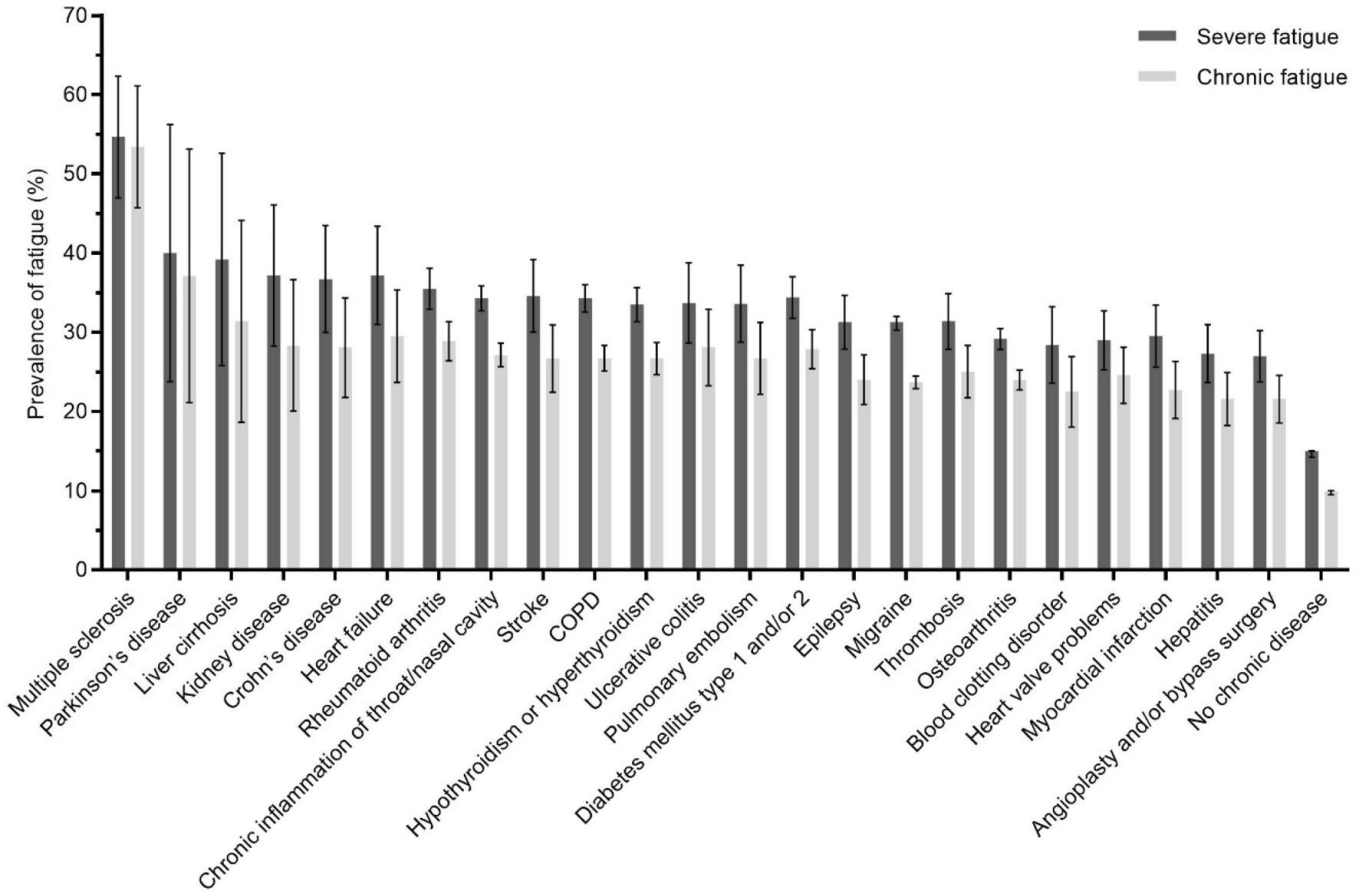
Prevalence and 95% Confidence Intervals of severe and chronic fatigue across 23 chronic diseases and in subjects without a chronic disease. Of note: (1) subjects that did not report their fatigue duration were omitted from the calculation of chronic fatigue, (2) the bars represent participants with multi-morbidity, hence subjects can be represented multiple times in the analyses. *COPD* chronic obstructive pulmonary disease.

Differentiating between the 23 included chronic diseases, prevalence rates ranged from 27 to 55% for severe fatigue and from 22 to 53% for chronic fatigue (Fig. 2). The 95% confidence intervals (95% CI’s) around the mean prevalence of severe and chronic fatigue of the included diseases generally overlap, except for MS which was significantly different from the other besides PD and liver disease. Table 2 describes the prevalence rates of severe and chronic fatigue among the classes of chronic medical conditions (Table 2).

**Table 2:** Prevalence of severe and chronic fatigue in classes of chronic medical conditions and no chronic disease.

	n	Severe fatigue n(%)	Chronic fatigue n(%)
**Neurological diseases**			
Including migraine	15,505	3882 (25.0)	2855 (18.4)
Excluding migraine	1672	482 (28.8)	369 (22.1)
Respiratory diseases	7652	2097 (27.4)	1574 (20.6)
Rheumatic diseases	7526	1765 (23.5)	1327 (17.6)
Endocrine and metabolic diseases	4142	1086 (26.2)	808 (19.5)
Circulatory diseases	3358	756 (22.5)	553 (16.5)
Liver diseases	873	185 (21.2)	138 (15.8)
Inflammatory bowel diseases	676	185 (27.4)	143 (21.2)
Blood diseases	442	98 (22.2)	76 (17.2)
Kidney disease	144	45 (31.3)	32 (22.2)
No chronic disease	47,324	6876 (14.5)	4616 (9.8)

*The classes of chronic medical conditions represent participants with multi-morbidity, hence subjects can be presented multiple times in the analyses.

### Impact of multi-morbidity on severe and chronic fatigue compared to participants without a chronic disease

**Figure 3:**
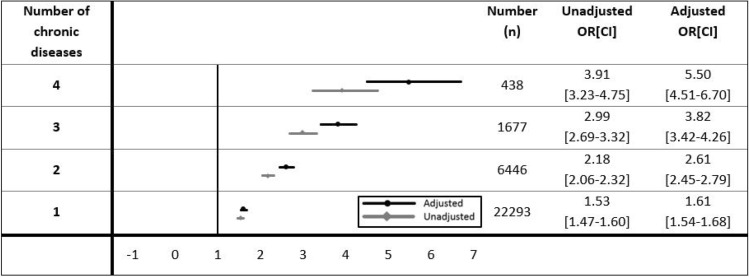
Unadjusted and adjusted odds ratios and 95% CI of experiencing severe fatigue for participants with one to four chronic diseases compared to participants without a chronic disease. Reference = participants without a chronic disease. Adjusted odds ratio corrected for age and sex.

The adjusted odds ratio [95% CI] of having severe fatigue was 1.6 [1.5–1.7], 2.6 [2.5–2.8], 3.8 [3.4–4.3], and 5.5 [4.5–6.7] for one, two, three, and four chronic diseases compared to having no chronic disease, respectively (Fig. 3). Similar results were found for chronic fatigue (1.5 [1.5–1.6], 2.3 [2.2–2.5], 3.2 [2.8–3.6], and 4.9 [3.9–6.1] for one, two, three, and four chronic diseases respectively, see Online Supplemental Fig. 2). Note that the 95%CI intervals did not overlap, showing that the likelihood of having severe and chronic fatigue significantly increased with having multiple chronic diseases.

### The disease-specific or trans-diagnostic relationship between fatigue severity and the predisposing and associated factors

The trans-diagnostic *versus* generic ANOVA model resulted in a statistically significant main effect of the presence of chronic disease, *F*(1, 69,007) = 691.306, *p* < 0.001 (model A, Online Supplemental Table 1). Adding the possible predisposing and associated variables, chronic disease remained a statistically significant predictor of fatigue severity *F*(1, 44,451) = 315.533, *p* < 0.001, though the *F* value decreased (model B, Online Supplemental Table 1). Similar results were found for the specific type of chronic disease (*F*(14, 21,670) = 10.660, *p* < 0.001 and *F*(14, 13,526) = 2.567, *p* = 0.001, respectively) (Table 3, model A and B).

**Table 3:** The disease-specific or trans-diagnostic relationship between fatigue severity and the predisposing and associated factors.

Disease-specific *versus* trans-diagnostic model	Model A		Model B		Model C	
	F(df)	*p* value	F(df)	*p* value	F(df)	*p* value
Specific chronic disease*	10.660 (14)	< 0.001	2.567 (14)	0.001	0.814 (14)	0.655
**Sex**			**7.539 (1)**	**0.006**	0.909 (1)	0.340
**Education level**			**34.694 (2)**	** < 0.001**	3.267 (2)	0.038
**Household composition**			**7.286 (4)**	** < 0.001**	0.330 (4)	0.858
Partner			0.964 (1)	0.326	1.986 (1)	0.159
Work situation			2.182 (1)	0.140	3.514 (1)	0.061
**Smoking**			**5.228 (1)**	**0.022**	0.266 (1)	0.606
**Depressive disorder**			**324.275 (1)**	** < 0.001**	64.908 (1)	< 0.001
**Anxiety disorder**			**434.870 (1)**	** < 0.001**	53.645 (1)	< 0.001
**Bodily pain that hampers in performing activities**			**394.341 (1)**	** < 0.001**	66.264 (1)	< 0.001
**Leisure-time sports activities**			**302.561 (1)**	** < 0.001**	64.434 (1)	< 0.001
**Age**			**225.963 (1)**	** < 0.001**	46.319 (1)	< 0.001
**BMI**			**108.504 (1)**	** < 0.001**	15.192 (1)	< 0.001
Diastolic blood pressure			2.607 (1)	0.106	2.446 (1)	0.118
**Resting heart rate**			**22.245 (1)**	** < 0.001**	20.845 (1)	< 0.001
**Neuroticism facets of anger/hostility, self-consciousness, impulsivity, and vulnerability**			**865.091 (1)**	** < 0.001**	148.417 (1)	< 0.001
No. of people in household			1.486 (1)	0.223	1.725 (1)	0.189
Disease × sex					0.923 (14)	0.532
Disease × education level					1.049 (28)	0.395
Disease × household composition					0.935 (54)	0.610
Disease × partner					0.950 (13)	0.499
Disease × work situation					1.351 (14)	0.168
Disease × smoking					1.343 (14)	0.173
**Disease x depressive disorder**					**1.834 (14)**	**0.029**
**Disease x anxiety disorder**					**1.785 (14)**	**0.035**
Disease × bodily pain that hampers in performing activities					1.015 (14)	0.435
Disease × leisure-time sports activities					0.362 (14)	0.985
Disease × age					1.087 (14)	0.364
Disease × BMI					1.060 (14)	0.390
Disease × diastolic blood pressure					1.364 (14)	0.162
Disease × resting heart rate					1.505 (14)	0.100
Disease × neuroticism facets of anger/hostility, self-consciousness, impulsivity, and vulnerability					0.741 (14)	0.734
Disease × No. of people in household					0.506 (14)	0.931

*no*. number, *df* degrees of freedom, *BMI* body mass index

*The factor ‘specific chronic disease’ reflects the participants with single-morbidity, entered as a categorical variable with 15 categories (each category reflecting a specific chronic disease with no intrinsic order). Model A: explores the impact of the main effect of ‘specific chronic disease’ on fatigue severity. Model B: investigates the main effects of ‘specific chronic disease’ and the predisposing and associated factors (predictors) on fatigue severity. Model C: studies the impact of the main effects plus the interaction effect between ‘specific chronic disease’ and the predisposing and associated factors on fatigue severity.

The following factors did not show a significant interaction effect with the type of chronic disease, but did have a significant main effect on fatigue severity (model B, Table 3): female gender, lower education level, living in a single parent household, current smoker, bodily pain that hampers in performing activities, no leisure-time sports activities, younger age, higher BMI, increased resting heart rate, and having higher scores on the neuroticism domain (similar results were found for the separate facets of anger/hostility, self-consciousness, impulsivity, and vulnerability, data not shown). The main effects of the type of chronic disease and the possible predisposing and associated factors explained 26% (adjusted R^2^) of the variance in fatigue (model B, Table 3).

Statistically significant interaction effects were found between chronic disease and the presence of a depressive- or anxiety disorder (p < 0.05, model C, Table 3), implying that the relationship between the presence of a depressive- or anxiety disorder and fatigue severity was influenced by the type of chronic disease. In specific, the linear regression analyses indicated that having a depression was associated with more severe fatigue for all included chronic diseases, though the size of the standardized coefficient differed between the chronic diseases (from β = 0.19 in participants with blood clotting disorder to β = 0.43 in participants with heart valve problems). Having an anxiety disorder was associated with increased fatigue severity for all chronic diseases except for blood clotting disorder and stroke (Online Supplemental Table 2).

### The trans-diagnostic or generic relationship between fatigue severity and the predisposing and associated factors

The trans-diagnostic *versus* generic ANOVA model indicated that none of the factors, apart from being currently employed and not being involved in leisure time sports activities, showed significant interaction effects with chronic disease (model C, Online Supplemental Table 1). This suggests that these factors are generic predisposing and associated factors of fatigue both in participants with and without a chronic disease. A post-hoc analysis, comparing the prevalence of the predisposing and associated factors between participants with and without a chronic disease, showed that all the generic factors that were associated with fatigue were significantly more often present or more pronounced in participants with chronic disease(s) (Table 1).

### Predisposing and associated factors of clinically relevant levels of severe and chronic fatigue in participants with single- and multi-morbidity

In addition, logistic regression analyses were performed to investigate whether the predisposing and associated factors were related to clinically relevant levels of fatigue (e.g. severe and chronic fatigue) in participants with single- and multi-morbidity (Online Supplemental Table 3). Similar factors as in the ANOVA models (Table 3) were found to increase the likelihood of experiencing severe fatigue. Yet, smoking status was not significantly associated with severe fatigue, whereas being unemployed and having a lower DBP were. In addition, an inverse association compared to the ANOVA model was found for education level (e.g. the likelihood for experiencing severe fatigue was significantly increased for higher instead of lower educated people) (model A, Online Supplemental Table 3). Similar results were found for the likelihood of being chronically fatigued (model B, Online Supplemental Table 3), though being a female and being a single parent were not significantly associated with chronic fatigue.

## Discussion

The present study is to the best of our knowledge the first to report prevalence rates of severe and chronic fatigue in a population-based cohort among a wide range of conditions using a validated fatigue questionnaire. The results showed that severe and chronic fatigue are common in chronic disease. The prevalence of severe and chronic fatigue was significantly higher in participants with chronic disease compared to healthy subjects. Moreover, direct comparison of prevalence rates of fatigue between chronic diseases showed remarkably similar prevalence rates of severe and chronic fatigue across chronic diseases, except for MS which was significantly higher from the other diseases besides PD and liver disease. The current study furthermore shows the impact of multi-morbidity on severe and chronic fatigue. With each additional chronic disease, the likelihood of having severe and chronic fatigue was found to increase significantly. This finding warrants the attention of healthcare professionals and policy makers, as the proportion of patients living with multiple chronic conditions rises due to the ageing population^11,12^.

The current study confirms that chronic disease is associated with more severe and chronic fatigue. However, most participants with a chronic disease do not experience severe or chronic fatigue. We identified several factors associated with fatigue severity in chronic disease: female gender, younger age, having a lower education level, living in a single parent household, having higher scores on neuroticism facets of anger/hostility, self-consciousness, impulsivity, and vulnerability, being a current smoker, having a higher BMI, increased resting heart rate, bodily pain that hampers in performing activities, and not being involved in leisure-time sports activities. Having a depressive disorder was also found to be associated with more severe fatigue in chronic disease, though the strength of the standardized coefficient differed among chronic diseases. This finding is in line with that of a previous study that found the same link between several factors associated with fatigue in chronic disease, albeit with a different strength^16^. Anxiety disorder was associated with fatigue severity in all chronic diseases, except in blood clotting disorder and stroke. In contrast, previous studies performed in post stroke patients indicated that anxiety is a common symptom, which is associated with fatigue^58,59^. The differences to the current findings may be attributed to the smaller sample sizes of these patient groups.

To date, the abovementioned predisposing and associated factors have often been studied in a disease-specific context^6,21–32,37–39,60–62^. Thus far, only one study investigated whether fatigue is a disease-specific or trans-diagnostic symptom in chronic disease^16^. In that study, female gender, younger age, pain and reduced physical activity were trans-diagnostically associated with fatigue severity. Reduced motivational and concentration problems, sleep disturbances, lower levels of physical functioning and lower self-efficacy concerning fatigue, which have not been evaluated in the current study, were also associated with fatigue severity across diseases. However, to the best of our knowledge the current study is the first to indicate that the predisposing and associated factors of fatigue are not only trans-diagnostic, but also generic. With the exception of employment status and being involved in sports activities, all factors were associated with fatigue in participants with a chronic disease and in healthy subjects to the same extent. Nevertheless, even though the predisposing and associated factors seem to be generic, the observation that the identified factors were more pronounced or more prevalent in chronic disease might partly explain the higher prevalence of fatigue in subjects with a chronic disease. Moving away from a disease-specific to a trans-diagnostic, or even generic focus on fatigue may help to accelerate the development of interventions for fatigue to improve daily functioning of patients with a chronic disease. That is, findings from fatigue research in one chronic disease can be generalized to other chronic diseases. Moreover, a trans-diagnostic approach also supports the complexity of multi-morbid chronic disease management, as disease-centered treatments will not fully address the comprehensive needs of patients with multiple chronic diseases. To date, the effectiveness of cognitive behavioral therapy and exercise therapy in reducing fatigue has been demonstrated in specific chronic diseases such as RA, T1DM, MS, COPD, and end-stage renal disease^63–68^. These interventions target factors such as physical activity which has been found to be a trans-diagnostic factor for fatigue and may, therefore, be effective for multiple chronic diseases (i.e. trans-diagnostic disease management).

Several limitations have to be considered when interpreting the results. First, the assessment of chronic disease was based on self-report which may be unreliable. Second, participants with a chronic disease are perhaps less likely to participate in studies such as Lifelines, which might have led to an underrepresentation of participants with a chronic disease, and therefore severe and chronic fatigue. Also, the exclusion of participants with low life expectancy might have biased the results. However, significant bias seems unlikely, as the prevalence rates of the chronic diseases in our study are comparable to those in the Dutch general population (Statistics Netherlands, CBS). Nevertheless, an underrepresentation of chronic diseases may also be true for general population statistics, as this is likewise based on self-report. Third, the development of new chronic diseases was re-evaluated during the first follow-up measurement. Nevertheless, not in the same manner as during baseline. Therefore, it was decided to only use the baseline information on chronic disease. Hence, no information was available about the development of new chronic diseases in the years between this assessment and the evaluation of fatigue. Also, the list of chronic diseases may not be comprehensive, as the chronicity of certain diseases (e.g., asthma) could not be determined. In addition, the measurement of fatigue may be subject to recall bias as it is does not capture diurnal variations of fatigue and is not measured in real time. Fourth, participants who were unable to understand the Dutch language were not considered eligible, which might have led to an underrepresentation of certain ethnicity groups. Fifth, the time-independent predisposing factors were not corrected for baseline fatigue, as these data were not available. It should be noted that no firm conclusions can be drawn on the direction of the association between the associated factors and fatigue. A cohort study design in which patients free of fatigue are followed over time, would provide us with in-depth information about the direction of the causality and would account for the non-response bias of patients with more profound fatigue or chronic diseases. Sixth, one should keep in mind that the current study only took a limited number of predisposing and associated factors of fatigue into account which explained 26% of the variance in fatigue. Future studies are needed to further unravel the underlying factors of fatigue in chronic disease that possibly can be addressed in interventions, thereby evaluating biological mechanisms like alterations in the hypothalamic–pituitary–adrenal (HPA) axis, autonomic nervous system, or inflammatory markers^69^. Then again, it should be evaluated whether these biological mechanisms are disease-specific, trans-diagnostic, or generic. Moreover, factors such as fatigue related beliefs^20^ which have been reported to be associated with fatigue in chronic disease should be considered.

In conclusion, the high prevalence rates of severe and chronic fatigue in chronic disease emphasize the clinical relevance of assessing fatigue in patients with a chronic disease. Screening for fatigue is of particular importance in patients with multi-morbidity, as the results of the current study indicate that the severity of fatigue increases in patients with multiple chronic diseases. Moreover, it appears that the predisposing and associated factors of fatigue are likely to be trans-diagnostic or even (mostly) generic for the general population. Several, predisposing and associated factors were identified. This provides direction to the development of trans-diagnostic interventions for fatigue and has also clinical implications for multi-morbid chronic disease management. Future studies are needed to further examine the underlying factors of fatigue in chronic disease and evaluate trans-diagnostic interventions in randomized clinical trials in samples with patients with different chronic diseases.

## Supplementary Information

Supplementary Information.

## Data Availability

We are not permitted to share individual data from the Dutch Lifelines study. Information on applying for access to the Dutch Lifelines data is available at https://www.lifelines.nl/researcher/how-to-apply.
