# Supplementary material for: Fatigue in patients with chronic disease: results from the population-based Lifelines Cohort Study
Source: Sci Rep. 2021 Oct 25;11:20977. doi: 10.1038/s41598-021-00337-z (PMC8546086; doi:10.1038/s41598-021-00337-z)
Supplement: Supplementary file 1 — Supplementary Information. [file 41598_2021_337_MOESM1_ESM.pdf]

Online Supplement

Fatigue in patients with chronic disease: results from the population-based Lifelines cohort study

Yvonne MJ Goërtz[1,2,3*], Annemarie MJ Braamse[4], Martijn A Spruit[1,2,3], Daisy JA Janssen[1,5], Zjala Ebadi[6], Maarten Van Herck[1,2,3,7], Chris Burtin[7], Jeannette B Peters[8] , Mirjam AG Sprangers[4], Femke Lamers[9], Jos WR Twisk[10], Melissa SY Thong[4], Jan H Vercoulen[6], Suzanne E Geerlings[11], Anouk W Vaes[1], Rosanne JHCG Beijers[2,3], Martijn van Beers[2,3]Annemie MWJ Schols[2,3], Judith GM Rosmalen[12], Hans Knoop[4]

[1]Department of Research and Development, Ciro, Horn, the Netherlands
[2]NUTRIM School of Nutrition and Translational Research in Metabolism, Maastricht, the Netherlands
[3]Department of Respiratory Medicine, Maastricht University Medical Centre (MUMC+), Maastricht, the Netherlands
[4]Department of Medical Psychology, Amsterdam University Medical Centers, Amsterdam Public Health Research Institute, University of Amsterdam, Amsterdam, the Netherlands
[5]Department of Health Services Research, Care and Public Health Research Institute, Faculty of Health, Medicine and Life Sciences, Maastricht University, Maastricht, the Netherlands.
[6]Radboud university medical center, Radboud Institute for Health Sciences, Department of Medical Psychology, Nijmegen, The Netherlands.
[7]REVAL – Rehabilitation Research Center, BIOMED – Biomedical Research Institute, Faculty of Rehabilitation Sciences, Hasselt University, Diepenbeek, Belgium
[8]Radboud university medical center, Radboud Institute for Health Sciences, Department of Lung diseases, Nijmegen, The Netherlands.
[9]Amsterdam UMC, Vrije Universiteit, Department of Psychiatry, Amsterdam Public Health Research Institute, Amsterdam, the Netherlands
[10]Amsterdam UMC, Vrije Universiteit Amsterdam, Department of Epidemiology and Biostatistics, Amsterdam Public Health research institute, Amsterdam, the Netherlands
[11]Department of Internal Medicine, Amsterdam University Medical Centers, University of Amsterdam, Amsterdam, the Netherlands
[12]University of Groningen, University Medical Center Groningen, Departments of Psychiatry and Internal Medicine, Groningen, the Netherlands

*Corresponding author

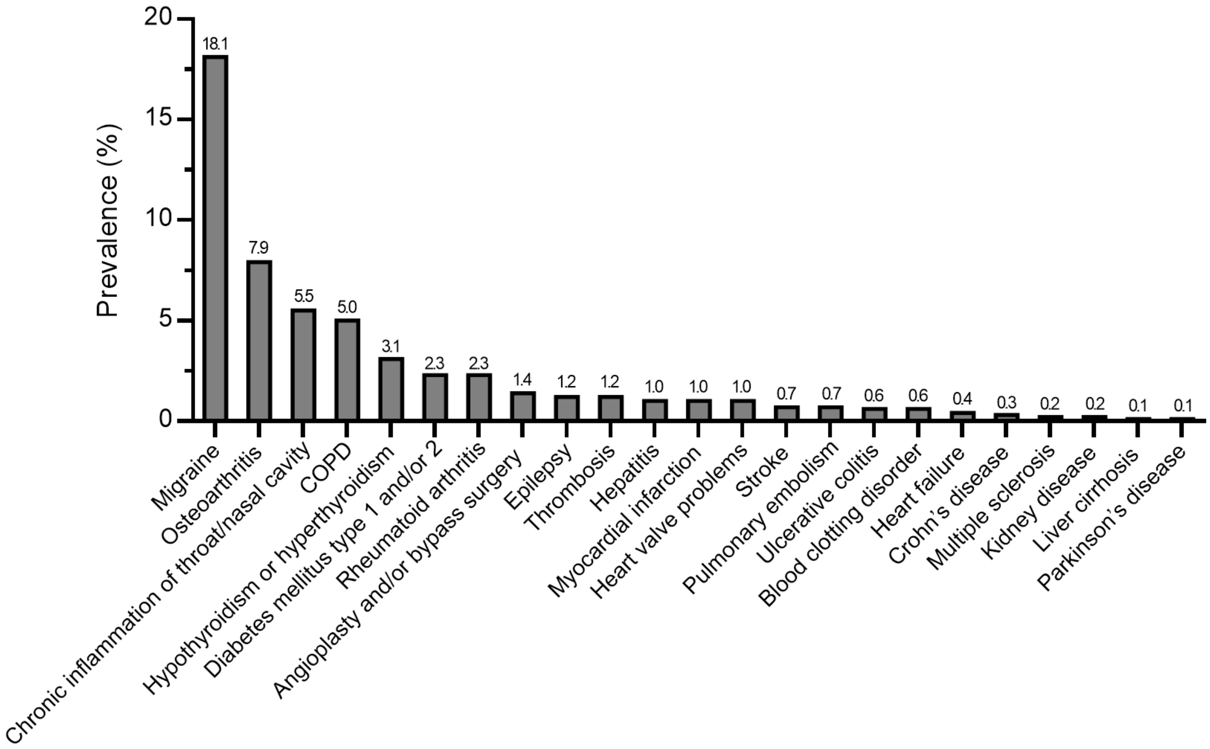

Online Supplement Figure 1: Prevalence of chronic diseases in the total sample (n=78,363)

Online Supplement Figure 1 presents the prevalence of chronic diseases in the sample. The most common chronic condition is migraine (18%), followed by osteoarthritis (8%), chronic inflammation of the throat/nasal cavity (6%), COPD (5%) and hypothyroidism or hyperthyroidism (3%). PD and liver cirrhosis were the least common disease in the cohort (0.1%). More than half (60%, n=47,324) of the total sample had no chronic disease, whereas 28% (n=22,293) had one chronic disease (single-morbidity) and 11% (n=8,746) had multiple chronic diseases concurrently.

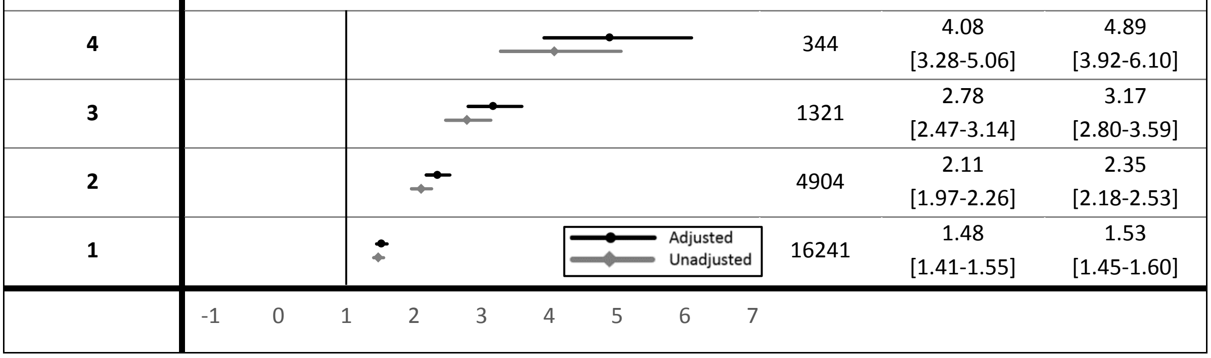

Number of chronic diseases	Number (n)	Unadjusted OR[CI]	Adjusted OR[CI]
**4**	344	4.08 [3.28-5.06]	4.89 [3.92-6.10]
**3**	1321	2.78 [2.47-3.14]	3.17 [2.80-3.59]
**2**	4904	2.11 [1.97-2.26]	2.35 [2.18-2.53]
**1**	16241	1.48 [1.41-1.55]	1.53 [1.45-1.60]

Online Supplement Figure 2: Unadjusted and adjusted odds ratios and 95%CI of experiencing chronic fatigue for participants with one to four chronic diseases compared to participants without a chronic disease. Reference = participants without a chronic disease. Adjusted odds ratio corrected for age and sex.

Online Supplement Table 1: The trans-diagnostic or generic relationship between fatigue severity and the predisposing and associated factors

Trans-diagnostic *versus* generic model	Model A		Model B		Model C	
	F(df)	*p*-value	F(df)	*p*-value	F(df)	*p*-value
**Chronic disease *versus* no chronic disease**	691.306(1)	<0.001	315.533(1)	<0.001	4.642(1)	0.031
**Sex**			**18.793(1)**	**<0.001**	16.367(1)	<0.001
**Education level**			**95.460(2)**	**<0.001**	87.494(2)	<0.001
**Household composition**			**27.479(4)**	**<0.001**	21.760(4)	<0.001
Partner			0.137(1)	0.712	0.064(1)	0.801
**Work situation**			**4.241(1)**	**0.039**	0.852(1)	0.356
**Smoking**			**28.555(1)**	**<0.001**	21.295(1)	<0.001
**Depressive disorder**			**1001.169(1)**	**<0.001**	938.841(1)	<0.001
**Anxiety disorder**			**1392.900(1)**	**<0.001**	1267.694(1)	<0.001
**Bodily pain that hampers in performing activities**			**1013.220(1)**	**<0.001**	988.351(1)	<0.001
**Leisure-time sports activities**			**827.905(1)**	**<0.001**	761.893(1)	<0.001
**Age**			**771.708(1)**	**<0.001**	644.277(1)	<0.001
**BMI**			**322.708(1)**	**<0.001**	299.008(1)	<0.001
**Diastolic blood pressure**			**5.073(1)**	**0.024**	5.513(1)	0.019
**Resting heart rate**			**100.679(1)**	**<0.001**	81.268(1)	<0.001
**Neuroticism facets of anger/hostility, self-consciousness, impulsivity, and vulnerability**			**3144.863(1)**	**<0.001**	2680.557(1)	<0.001
**No. of people in household**			**7.118(1)**	**0.008**	6.136(1)	0.013
Disease x Sex					0.049(1)	0.825
Disease x Education level					1.292(2)	0.275
Disease x household composition					0.724(4)	0.575
Disease x Partner					2.331(1)	0.127
**Disease x Work situation**					**12.515(1)**	**<0.001**
Disease x Smoking					0.385(1)	0.535
Disease x Depressive disorder					1.945(1)	0.163
Disease x Anxiety disorder					1.295(1)	0.255
Disease x Bodily pain that hampers in performing activities					0.086(1)	0.770
**Disease x Leisure-time sports activities**					**6.367(1)**	**0.012**
Disease x Age					1.806(1)	0.179
Disease x BMI					0.423(1)	0.515
Disease x Diastolic blood pressure					0.379(1)	0.538
Disease x Resting heart rate					0.434(1)	0.510

Disease x Neuroticism facets of anger/hostility, self-consciousness, impulsivity, and vulnerability					1.193(1)	0.275
Disease x No. of people in household					0.184(1)	0.668

** The factor 'chronic disease versus no chronic disease' reflects the participants with- and without chronic disease, coded as 0=no chronic disease, 1 = single-morbidity. Model A: explores the impact of the main effect of 'chronic disease versus no chronic disease' on fatigue severity. Model B: investigates the main effects of 'chronic disease versus no chronic disease' and the independent variables (predictors) on fatigue severity. Model C: studies the impact of the main effects plus the interaction effect between 'chronic disease versus no chronic disease' and the independent variables on fatigue severity. Abbreviations: no. = number; df = degrees of freedom, BMI = body mass index.*

Online Supplement Table 2: Interaction effect results between the presence of a depressive- or anxiety disorder and fatigue

	Depression				Anxiety			
	df	Unstandardized Coefficient	Standardized coefficient	95% CI	df	Unstandardized coefficient	Standardized coefficient	95% CI
COPD	1488	16.875	0.306	14.208 – 19.542*	1488	12.257	0.286	10.167 – 14.348*
Blood clotting disorder	132	13.074	0.189	1.308 – 24.841*	132	1.745	0.050	-4.238 – 7.728
Stroke	143	9.978	0.196	1.686 – 18.270*	143	1.640	0.032	-6.812 –10.091
Angioplasty and/or bypass surgery	158	17.013	0.220	5.106 – 28.920*	158	17.000	0.308	8.707 – 25.293*
Ulcerative colitis	181	21.737	0.342	12.967 – 30.507*	181	13.205	0.235	5.156 – 21.254*
Thrombosis	215	18.236	0.416	12.865 – 23.607*	215	11.970	0.325	7.276 – 16.665*
Heart valve problems	245	20.425	0.429	14.998 – 25.852*	245	9.326	0.202	3.630 – 15.022*
Hepatitis	294	23.215	0.417	17.404 – 29.027*	294	16.482	0.336	11.177 – 21.786*
Epilepsy	387	15.058	0.250	9.210 –20.906*	387	10.306	0.246	6.243 – 14.368*
Rheumatoid arthritis	546	18.726	0.318	14.027 – 23.425*	546	13.117	0.273	9.228 – 17.006*
Diabetes mellitus type 1 and/or 2	597	17.786	0.321	13.566 – 22.007*	597	12.639	0.292	9.308 – 15.970*
Hypothyroidism or hyperthyroidism	945	17.118	0.290	13.514 – 20.722*	945	14.227	0.296	11.298 – 17.156*
Chronic inflammation of throat and/or nasal cavity	1731	13.677	0.260	11.280 – 16.075*	1731	12.176	0.333	10.533 – 13.800*
Osteoarthritis	2356	18.289	0.309	16.010 – 20.567*	2356	13.663	0.296	11.882 – 15.444*
Migraine	8224	16.302	0.302	15.190 –17.414*	8224	12.440	0.325	11.658 – 13.222*

Abbreviations: df = degrees of freedom; CI = Confidence Interval. *Asteriks indicates a p≤0.05.

Online Supplement Table 3: Binary logistic regressions

	Model A<br>*Severe fatigue*<br>*Included cases: 18,513*		*Model B*<br>*Chronic fatigue*<br>*Included cases: 14,246*	
	**OR[95%CI]**	***p*-value**	**OR[95%CI]**	***p*-value**
Chronic disease	1.62[1.49-1.76]	<0.001	1.54[1.40-1.69]	<0.001
Female gender	1.10[1.00-1.20]	0.05	0.91[0.82-1.01]	0.07
Age	0.98[0.97-0.98]	<0.001	0.98[0.98-0.99]	<0.001
Nr. of people in a household	0.96[0.90-1.02]	0.20	0.98[0.92-1.05]	0.63
Education MIDDLE	1.14[1.01-1.28]	0.03	1.08[0.95-1.23]	0.24
Education HIGH	1.35[1.19-1.54]	<0.001	1.21[1.04-1.40]	0.01
Partner	0.99[0.67-1.45]	0.94	1.08[0.71-1.65]	0.72
BMI	1.04[1.03-1.05]	<0.001	1.03[1.02-1.04]	<0.001
DBP	1.00[0.99-1.00]	0.02	0.99[0.99-1.00]	0.02
Resting heart rate	1.01[1.01-1.01]	<0.001	1.01[1.00-1.01]	<0.001
Work situation	0.83[0.76-0.91]	<0.001	0.70[0.63-0.78]	<0.001
Smoking status	1.07[0.96-1.18]	0.23	0.99[0.88-1.11]	0.85
Neuroticism facets of anger/hostility, elf-consciousness, impulsivity, and vulnerability	1.13[1.12-1.15]	<0.001	1.09[1.08-1.11]	<0.001
Depressive disorder	3.88[3.28-4.59]	<0.001	2.74[2.33-3.23]	<0.001
Anxiety disorder	2.94[2.61-3.31]	<0.001	2.34[2.07-2.64]	<0.001
Sports	0.58[0.54-0.63]	<0.001	0.55[0.51-0.60]	<0.001
Bodily pain that hampers in performing activities	4.14[3.59-4.78]	<0.001	3.10[2.67-3.61]	<0.001
Couple without children	0.87[0.58-1.29]	0.48	0.94[0.60-1.45]	0.77
Couple with children	1.03[0.67-1.57]	0.91	1.06[0.66-1.69]	0.82
Single parent	1.38[1.10-1.72]	0.01	1.26[0.98-1.61]	0.07
Other household composition	0.79[0.57-1.09]	0.15	0.85[0.59-1.23]	0.38

** Model A: dependent factor yes/no severe fatigue. Model B: dependent factor yes/no chronic fatigue. The independent factor "chronic disease" includes participants with single-morbidity versus multi-morbidity. Abbreviations: OR = Odds Ratio; CI = Confidence Interval; Nr. = number; BMI = Body Mass Index; DBP = Diastolic Blood Pressure.*
